# Supplementary material for: FERN – a Java framework for stochastic simulation and evaluation of reaction networks
Source: BMC Bioinformatics. 2008 Aug 29;9:356. doi: 10.1186/1471-2105-9-356 (PMC2553347; doi:10.1186/1471-2105-9-356)
Supplement: Additional file 1 — FERN distribution, Version 1.3. This archive contains the FERN source code and binaries as well as documentation and example models in FernML and SBML. [file 1471-2105-9-356-S1.zip › fern/doc/javadoc/fern/simulation/algorithm/AbstractBaseTauLeaping.html]

AbstractBaseTauLeaping


---


|  |  |  |
| --- | --- | --- |
| **Overview** \| **Package** \| **Class** \| **Use** \| **Tree** \| **Deprecated** \| **Index** \| **Help** | |  |
| PREV CLASS   **NEXT CLASS** | **FRAMES**    **NO FRAMES**     **All Classes** |  |
| SUMMARY: NESTED \| FIELD \| CONSTR \| METHOD | DETAIL: FIELD \| CONSTR \| METHOD |  |


---


## fern.simulation.algorithm Class AbstractBaseTauLeaping

```
java.lang.Object
  fern.simulation.Simulator
      fern.simulation.algorithm.GillespieEnhanced
          fern.simulation.algorithm.AbstractBaseTauLeaping
```

**Direct Known Subclasses:**: AbstractTauLeapingPropensityBoundSimulator, TauLeapingSpeciesPopulationBoundSimulator

---

``` public abstract class AbstractBaseTauLeaping extends GillespieEnhanced ```

Base class for all tau leaping procedures (which are different in the methods choosing
the timestep candidates for critical and noncritical reactions). It extends `GillespieEnhanced`
because it uses the SSP algorithm, when the timestep candidate is to small.

Each tau leaping algorithm only works with an `AbstractKineticConstantPropensityCalculator` and
only if the highest order of a reaction is maximal 3.

For references see Daniel T. Gillespie, Approximate accelerated stochastic simulation
of chemically reacting systems, Journal of chemical physics vol 115, nr 4 (2001); Cao et al., Efficient
step size selection for the tau-leaping simulation method, Journal of chemical physics 124, 044109 (2006)

**Author:**
:   Florian Erhard

**See Also:**: `GillespieEnhanced`

---

**Nested Class Summary**

**Nested classes/interfaces inherited from class fern.simulation.Simulator**

`Simulator.FireType`


| **Field Summary** | |
| --- | --- |
| `protected  Map<Integer,Integer>[]` | `productHistos` |
| `protected  Map<Integer,Integer>[]` | `reactantHistos` |
| `protected  boolean` | `verbose` |

**Fields inherited from class fern.simulation.algorithm.GillespieEnhanced**

`a_sum`

**Fields inherited from class fern.simulation.Simulator**

`a, stochastics, t`


**Constructor Summary**

`AbstractBaseTauLeaping(Network net)`
  
          Create the simulator for a given network.


| **Method Summary** | |
| --- | --- |
| `protected  double` | `chooseTauCriticals(BitVector criticals)`             choose the timestep candidate for the critical reactions |
| `protected abstract  double` | `chooseTauNonCriticals(BitVector criticals)`             choose the timestep candidate for the noncritical reactions by some method. |
| `double` | `getEpsilon()`             The epsilon is an error control parameter and bounds the expected change of the propensity functions by epsilon \* a\_sum. |
| `double` | `getLangevinThreshold()`             The Langevin threshold determines, when the normal distribution is used as an approximation for the poisson distribution. |
| `String` | `getName()`             Returns the name of this simulator |
| `int` | `getNCritical()`             The nCritical determines, when a reaction is called critical. |
| `int` | `getNumSimpleCalls()`             The numSimpleCalls determines, how often the SSA is called, when tau leaping is abandoned. |
| `double` | `getUseSimpleFactor()`             The useSimpleFactor determines, when the tau leaping is abandoned and the SSA method is used (if tau |
| `protected  int` | `getV(int species, int reaction)` |
| `boolean` | `isVerbose()`             Gets if each step is to print to stdout. |
| `void` | `performStep(SimulationController control)`             Performs a tau leaping step. |
| `void` | `setEpsilon(double epsilon)`             The epsilon is an error control parameter and bounds the expected change of the propensity functions by epsilon \* a\_sum. |
| `void` | `setLangevinThreshold(double langevinThreshold)`             The Langevin threshold determines, when the normal distribution is used as an approximation for the poisson distribution. |
| `void` | `setNCritical(int critical)`             The nCritical determines, when a reaction is called critical. |
| `void` | `setNumSimpleCalls(int numSimpleCalls)`             The numSimpleCalls determines, how often the SSA is called, when tau leaping is abandoned. |
| `void` | `setUseSimpleFactor(double useSimpleFactor)`             The useSimpleFactor determines, when the tau leaping is abandoned and the SSA method is used (if tau |
| `void` | `setVerbose(boolean verbose)`             Sets if each step is to print to stdout. |

**Methods inherited from class fern.simulation.algorithm.GillespieEnhanced**

`directMCTau, initialize, isEfficientlyAdaptSum, reinitialize, setAmount, setEfficientlyAdaptSum, setVolume`

**Methods inherited from class fern.simulation.Simulator**

`addObserver, fireReaction, fireReaction, getAmount, getAmountManager, getNet, getNextThetaEvent, getPropensity, getPropensityCalculator, getTime, getVolume, isInterpolateTheta, registerNewTheta, setInterpolateTheta, start, start, thetaEvent`

**Methods inherited from class java.lang.Object**

`clone, equals, finalize, getClass, hashCode, notify, notifyAll, toString, wait, wait, wait`

**Field Detail**

### verbose

```
protected boolean verbose
```

---


### reactantHistos

```
protected Map<Integer,Integer>[] reactantHistos
```

---


### productHistos

```
protected Map<Integer,Integer>[] productHistos
```


**Constructor Detail**

### AbstractBaseTauLeaping

```
public AbstractBaseTauLeaping(Network net)
```

:   Create the simulator for a given network.

    **Parameters:**: `net` - the simulation network


**Method Detail**

### chooseTauNonCriticals

```
protected abstract double chooseTauNonCriticals(BitVector criticals)
```

:   choose the timestep candidate for the noncritical reactions by some method.

    :   **Parameters:**: `criticals` - Bitvector identifying the critical reactions **Returns:**: timestep candidate for noncritical reactions

---


### chooseTauCriticals

```
protected double chooseTauCriticals(BitVector criticals)
```

:   choose the timestep candidate for the critical reactions

    :   **Parameters:**: `criticals` - Bitvector identifying the critical reactions **Returns:**: timestep candidate for critical reactions

---


### performStep

```
public void performStep(SimulationController control)
```

:   Performs a tau leaping step.
    First, critical reactions (that could exhaust one of its reactants) are idenfied.
    The threshold for criticals can be controlled by the field nCriticals.
    Second, a candidate timestep is chosen for noncritical reactions. If this timestep
    is to little (controlled by useSimpleFactor), `numSimpleCalls` steps
    from the SSP-Algorithm GillespieEnhanced are perform. Otherwise a second timestep
    candidate is drawn for the criticals (such that only one critical reaction is firing
    and only once in this leap). The smaller candidate is then used as tau.

    :   **Overrides:**: `performStep` in class `GillespieEnhanced`
    :   **Parameters:**: `control` - the simulators controller

---


### getV

```
protected int getV(int species,
                   int reaction)
```

---


### getLangevinThreshold

```
public double getLangevinThreshold()
```

:   The Langevin threshold determines, when the normal distribution is used as an
    approximation for the poisson distribution. The default value is Infinity (no
    approximation).

    :   **Returns:**: the langevinThreshold

---


### setLangevinThreshold

```
public void setLangevinThreshold(double langevinThreshold)
```

:   The Langevin threshold determines, when the normal distribution is used as an
    approximation for the poisson distribution. The default value is Infinity (no
    approximation).

    :   **Parameters:**: `langevinThreshold` - the langevinThreshold to set

---


### getUseSimpleFactor

```
public double getUseSimpleFactor()
```

:   The useSimpleFactor determines, when the tau leaping is abandoned and the SSA
    method is used (if tau:   **Returns:**: the useSimpleFactor

---


### setUseSimpleFactor

```
public void setUseSimpleFactor(double useSimpleFactor)
```

:   The useSimpleFactor determines, when the tau leaping is abandoned and the SSA
    method is used (if tau:   **Parameters:**: `useSimpleFactor` - the useSimpleFactor to set

---


### getNumSimpleCalls

```
public int getNumSimpleCalls()
```

:   The numSimpleCalls determines, how often the SSA is called, when tau leaping is
    abandoned. The default value is 100.

    :   **Returns:**: the numSimpleCalls

---


### setNumSimpleCalls

```
public void setNumSimpleCalls(int numSimpleCalls)
```

:   The numSimpleCalls determines, how often the SSA is called, when tau leaping is
    abandoned. The default value is 100.

    :   **Parameters:**: `numSimpleCalls` - the numSimpleCalls to set

---


### getNCritical

```
public int getNCritical()
```

:   The nCritical determines, when a reaction is called critical. It is critical, when
    the maximum number, a reaction can fire before exhausting one of its reactants
    is less than nCritical. The default value is 10.

    :   **Returns:**: the nCritical

---


### setNCritical

```
public void setNCritical(int critical)
```

:   The nCritical determines, when a reaction is called critical. It is critical, when
    the maximum number, a reaction can fire before exhausting one of its reactants
    is less than nCritical. The default value is 10.

    :   **Parameters:**: `critical` - the nCritical to set

---


### getEpsilon

```
public double getEpsilon()
```

:   The epsilon is an error control parameter and bounds the expected change of the
    propensity functions by epsilon \* a\_sum. It has to be 0 < epsilon << 1.
    The default value is 0.03.

    :   **Returns:**: the epsilon

---


### setEpsilon

```
public void setEpsilon(double epsilon)
```

:   The epsilon is an error control parameter and bounds the expected change of the
    propensity functions by epsilon \* a\_sum. It has to be 0 < epsilon << 1.
    The default value is 0.03.

    :   **Parameters:**: `epsilon` - the epsilon to set

---


### isVerbose

```
public boolean isVerbose()
```

:   Gets if each step is to print to stdout.

    :   **Returns:**: verbose

---


### setVerbose

```
public void setVerbose(boolean verbose)
```

:   Sets if each step is to print to stdout.

    :   **Parameters:**: `verbose` - verbose

---


### getName

```
public String getName()
```

:   Returns the name of this simulator

    :   **Overrides:**: `getName` in class `GillespieEnhanced`
    :   **Returns:**: simulator name


---


|  |  |  |
| --- | --- | --- |
| **Overview** \| **Package** \| **Class** \| **Use** \| **Tree** \| **Deprecated** \| **Index** \| **Help** | |  |
| PREV CLASS   **NEXT CLASS** | **FRAMES**    **NO FRAMES**     **All Classes** |  |
| SUMMARY: NESTED \| FIELD \| CONSTR \| METHOD | DETAIL: FIELD \| CONSTR \| METHOD |  |


---
